# Immunostimulatory Potential of Fruits and Their Extracts in Poultry

**DOI:** 10.3389/fimmu.2021.641696

**Published:** 2021-05-17

**Authors:** Teri-Lyn Hasted, Shayan Sharif, Patrick Boerlin, Moussa Sory Diarra

**Affiliations:** ^1^ Guelph Research and Development Center, Agriculture and Agri-Food Canada, Guelph, ON, Canada; ^2^ Department of Pathobiology, University of Guelph, Guelph, ON, Canada

**Keywords:** cranberry, blueberry, grape, other fruits, byproducts, poultry, immune stimulation

## Abstract

The impact of antibiotic use for growth promotion in livestock and poultry production on the rise of antimicrobial resistance (AMR) in bacteria led to the ban of this practice in the European Union in 2006 and a restriction of antimicrobial use (AMU) in animal agriculture in Canada and the United States of America. There is a high risk of infectious diseases such as necrotic enteritis due to *Clostridium perfringens*, and colibacillosis due to avian pathogenic *Escherichia coli* in antimicrobial-free broiler chickens. Thus, efficient and cost-effective methods for reducing AMU, maintaining good poultry health and reducing public health risks (food safety) are urgently needed for poultry production. Several alternative agents, including plant-derived polyphenolic compounds, have been investigated for their potential to prevent and control diseases through increasing poultry immunity. Many studies in humans reported that plant flavonoids could modulate the immune system by decreasing production of pro-inflammatory cytokines, T-cell activation, and proliferation. Fruits, especially berries, are excellent sources of flavonoids while being rich in nutrients and other functionally important molecules (vitamins and minerals). Thus, fruit byproducts or wastes could be important resources for value-added applications in poultry production. In the context of the circular economy and waste reduction, this review summarizes observed effects of fruit wastes/extracts on the general health and the immunity of poultry.

## Introduction

Poultry products are quickly becoming one of the most-consumed sources of protein around the world (1), making them very important to the global food supply. Globally, poultry meat demand and production are expected to continue rising more rapidly than any other types of meat. Current estimates predict that by 2026, poultry meat will account for 45% of global meat consumption. The increase in poultry production is especially apparent in developing countries, due to poultry’s status as an inexpensive and healthy meat with few cultural or religious barriers (2). Poultry products include meat from broiler chickens and turkeys, eggs from layers, and live chicks often exported to other countries, all of which are important parts of the agricultural sector. Unfortunately, as with any animals kept at high density, poultry are prone to infectious diseases. Farmers have a vested interest in keeping their flocks disease-free and growing faster and larger to meet consumers’ demands. Thus, antibiotics have been used for disease prevention and/or growth promotion in meat-type poultry farms. Due to the selection for and spread of antimicrobial resistance (AMR) genes among several bacterial species of food safety and public health concern, antimicrobial use (AMU) in animal agriculture is becoming increasingly restricted (3). Additionally, poultry pathogens carrying AMR genes can compromise the birds’ health due to increased difficulty in treating diseases associated with them.

Bacterial diseases, such as necrotic enteritis (NE), will continue to negatively impact poultry health and producers’ profits if they become harder to control in the future. As mentioned above, with many countries (including the United States and Canada) withdrawing, restricting, or banning AMU for growth promotion in poultry, producers are searching for new ways to protect their flocks from bacterial diseases that have thus far been managed with low doses of preventative antibiotics. There have been many proposed alternatives to antibiotics where vaccines are not available, including antimicrobial peptides, organic acids, enzymes, metals, clay, bacteriophages, prebiotics, probiotics, and phytochemicals (4). Some of these approaches are able to improve the growth of the chickens. For example, enzymes added to feed improve the digestibility and availability of nutrients in the feed, subsequently improving feed efficiency and growth. Other alternatives, such as antimicrobial peptides, modulate or restrict the population of gut microbiota (including potential pathogens), and increase the overall flock health by reducing the frequency of diseases. The administration of probiotics (*Lactobacillus* species or others) in the chickens’ diets has shown some success in both increasing growth rates and preventing diseases. Because the productivity of the chickens and their resistance to diseases are both important and linked to multitude of factors, there is no simple solution for replacing antibiotics. It is likely that none of the alternative approaches mentioned above alone would be effective enough, thus a combination of approaches would be more effective.

Current poultry feeds are formulated to meet the nutritional requirements of chickens, but more research is needed to determine if these feeds adequately support the bird’s immune system, or if additional ingredients are needed to prevent immune deficiencies. Ideally, boosting their immune function should help the birds to be more resistant to infectious diseases. Vaccines have been used in poultry production to prevent diseases; however, most of them target viral pathogens such as Marek’s disease virus, Newcastle disease virus, infectious bronchitis virus, and infectious bursal disease virus (5). Longer-lived layer hens typically receive almost all above mentioned vaccines, in addition to those against encephalomyelitis, fowlpox, and laryngotracheitis depending on production conditions and requirements (5). This vaccination schedule is standard in most areas of North America, but it can be modified to suit the surrounding disease environment.

Phytochemicals, particularly those found in certain fruit extracts, are high in antioxidant compounds, and have been shown to improve the immune systems and general health in humans (6, 7). It stands to reason, then, that fruits and their extracts could help prevent diseases by stimulating the immune system in poultry such as broiler chickens. The use of fruit wastes, whole fruits, and their derivatives as supplements to improve immunity is an attractive solution for both poultry farmers and fruit producers. Many studies have been conducted on fruits and their various products (juices, pomaces, extracts, and seeds) with regards to boosting immunity in humans (6, 8–10). Fewer studies have been done on their effects in poultry, or other livestock. Certain fruit products, such as cranberry and blueberry pomaces, have been reported to have antimicrobial activities against a variety of bacterial species (11, 12). Grape seed extracts have demonstrated effects on blood metabolites, the immune system, and overall productivity of broilers (13). Considering that fruit byproducts present an economical and value-added opportunity, they are worthy of further investigation as possible cost-effective alternative agents in poultry production. The application of optimized fruit products, along with proper husbandry and good antimicrobial stewardship, may contribute to healthier and more profitable poultry flocks, as well as decrease the need for antibiotics and therefore the spread of AMR. This review will focus primarily on the application of fruits, particularly berries, and their extracts to enhance poultry immunity.

## Immune System of Poultry

The avian immune system is complex and plays a critical role in protecting birds from infectious and metabolic diseases. Similar to the mammalian immune system, the avian immune system provides both innate and adaptive responses. The innate immune response is the first line of defense, responding non-selectively to a wide range of pathogens by phagocytizing and chemically attacking them. The adaptive immune system, consisting of both cellular and humoral immunity, is capable of launching a specific response to previously encountered pathogens. The adaptive immune response and its memory properties can be harnessed using vaccines to provide long-term immunity for birds against specific diseases. A detailed description of the avian immune system has been reported elsewhere (14) and will not be provided here.

A few factors pertinent to poultry farming must be noted. Mainly, broiler chickens live about six weeks, and have several immunological differences from laying hens, which live for 1-2 years. Since the bursa of Fabricius, the main site of B-cell development in birds, disappears by seven months of age, the bursa is always present in broilers but may be absent in older laying hens. Therefore, the bursa and bursa-body weight ratio has been used to assess relative immune health in broilers only (15). Another important practical difference is that broilers are vaccinated against a few prevalent and deadly diseases as stated above whereas layers are vaccinated against many more diseases (5). Heterophils, present throughout the lives of both types of birds, are important cells of the innate immune system, analogous to mammalian neutrophils and involved in the initial acute response to most pathogens (16). Unfortunately, some intracellular bacteria have evolved ways of avoiding phagocytosis and lysis by heterophils. In poultry, *Staphylococcus aureus* in particular has shown this ability which is troublesome as it can greatly impact the production of both broiler and layer flocks (17).

Cytokines are a diverse group of proteins or peptides secreted by cells to help direct both innate and adaptive immune responses. Cytokine levels are an indicator of the robustness of the immune system. Some cytokines in chickens can be used to evaluate stress levels, infections, or other immune functions. For example, chronically stressed chickens display elevated levels of interleukin-2 (IL-2) and granulocyte colony stimulating factor (G-CSF) in spleen tissue, and IL-1β, IL-2, IL-18, interferon gamma (IFN-γ), and IL-6 in thymic tissue (18). Chickens orally challenged with *Salmonella enterica* serovar Typhimurium showed high levels of IL-1β, IL-8, and other cytokines in intestinal and liver tissues, but not in the spleen (19). IL-10 plays an important role in the ceca, and has higher expression in birds susceptible to *Eimeria tenella* (20). A co-infection with both *Eimeria* spp. and *C. perfringens* has been shown to downregulate the production of IFN-α, IFN-γ, IL-1β, IL-2, IL-12, IL-13, IL-17 and transforming growth factor-beta 4 (TGF-β4) (21). Additionally, transcript analysis showed T helper 1 (Th1) interleukins such as IFN-γ, IL-2, and IL-12p40 (primarily mediators of cellular immunity) and T helper 2 (Th2) interleukins such as IL-4, IL-5, and IL-10 (primarily mediators of humoral immunity) were upregulated in response to challenge with infectious bursal disease virus in four-week old white leghorn chicks (22). Cytokine responses vary depending on the chicken line, the type of tissue assayed, the type of infection (bacterial, parasitic, viral), and the post-infection time (23, 24). Specifically, Ross broiler chickens showed significantly more expression of avian β-defensins in response to NE when compared to Cobb broilers (23). It is worthwhile to note that different broiler lines can have different immune responses to the same challenge conditions, even if this particular reaction may not be the same across all types of infections. Although a few recent studies have been conducted on cytokine modulation in response to fruit supplementation in chickens, more research and better standardization could elucidate quantitative effects of fruit byproducts on the immune system.

### Modulation of the Poultry Immune System

Immune response can be evaluated in a variety of ways, such as quantifying leukocyte populations, cytokine responses, antibody concentrations, inflammation markers, phagocytic capabilities, and the masses of various immune organs. A multitude of strategies have been employed to enhance the immune and inflammatory responses in poultry. The most successful of these strategies have been vaccinations, which take advantage of the memory property of the adaptive immune system. However, there are many diseases for which no vaccine currently exists, or existing vaccines are in need of improvement. Many provide suboptimal protection, require adjuvants, and may be tedious and expensive to administer (25). Vaccines against bacterial diseases, such as NE, have lagged behind, due to the complexity of these diseases and the counterincentive of efficient and cost-effective antibiotics. Some new vaccines have been developed using attenuated *Salmonella* as a vector, but these must be administered *via* oral gavage and are therefore time-consuming in a large-scale regular production cycle (26).

Besides vaccinations to incite immunity against a specific pathogen, immunostimulating products or compounds to increase general non-specific immunity, have been also suggested as alternative solutions in poultry. These products may have pleiotropic actions on the host’s immune system. Accordingly, probiotics have been proposed as immunomodulators, to reduce pro-inflammatory cytokine expression in the gut, and increase the IgA secretion and the integrity of the gut epithelial barrier, and the serum antibody levels to certain antigens (4, 27–30). Probiotics have been specifically shown to decrease the expression of IL-12 and IFN-γ resulting from subsequent infection with *S.* Typhimurium (31). Another study reported that different strains of *Lactobacillus* can have different effects on spleen and caecal tonsil mononuclear cells: specifically that *L. acidophilus* induces primarily Th1 cytokines, while *L. salivarius* is capable of inducing more anti-inflammatory cytokines (32). Antimicrobial peptides (specifically rabbit sacculus rotundus antimicrobial peptides) have also been employed, which act both directly on bacteria and as immunostimulants leading to increased IgA levels, mast cell populations and intraepithelial lymphocyte counts (33). However, antimicrobial peptides are not an ideal solution due to their high production costs and the ability of bacteria to eventually develop resistance against them. Heterophil levels, and subsequently pro-inflammatory cytokine levels, have been artificially increased by orally administering ulvan, an extract of sea lettuce (*Ulva armoricana*), a proposed agonist of Toll-Like Receptor (TLR) 2 and 4 (34).

## Fruit Byproducts: Processing and Properties

**Figure 1:**
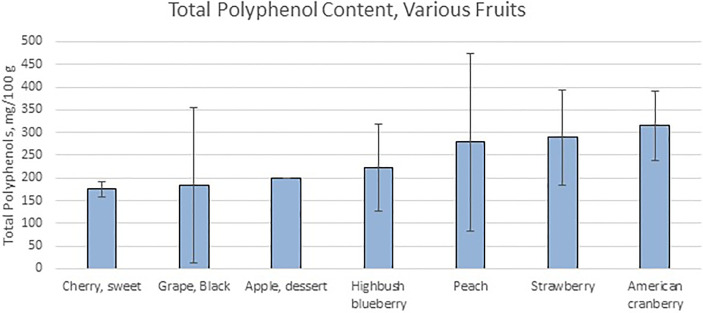
Total Polyphenol Content of Various Fruits shows the values of total polyphenol measured *via* Folin assay by various research groups. Data from (35).

Fruit byproducts are complex mixtures made from processing whole fruit, seeds, juices, or pomace using a variety of techniques. Additional processing of these byproducts can result in the generation of fruit extracts, generally by using a liquid solvent to extract and concentrate valuable chemical components. These processing techniques could include freeze drying, extrusion, separation of fractions *via* solvent/water extraction, or dialysis to prepare pure extracts. The most abundant phenolic compounds present in fruits relevant to this paper can be found in **Table 1** (35), although this does not represent an exhaustive list, as it omits trace compounds and non-phenolic compounds thought to have effects on bacteria. Fruit pomaces and extracts can contain flavonols (such as quercetin, kaempferol, myricetin and isorhamnetin), flavonoids (such as anthocyanins), flavanols (such as catechins, epicatechins, and procyanidins), flavanones, proanthocyanidins, or gallocatechins (prodelphinidins), some of which are involved in the process of fruit browning (36, 37). The non-phenolic contents such as dietary fibers, proteins, oligosaccharides, vitamins, and minerals, contain different chemical constituents depending on the species or subspecies of fruit, extraction process, and the nature of the solvent used. Freeze-drying and solvent extraction methods were applied in combination to organic cranberry and wild blueberry pomaces, after which their polyphenolic profiles were analyzed using a combination of the Glories method and the polyvinylpolypyrrolidone (PVVP) method, and high levels of polyphenols, particularly anthocyanins as well as antioxidants, were found (38). Alternatively, using high temperatures and water as a solvent when extracting grape byproducts resulted in extracting more flavonoids and procyanidins, whereas an organic solvent provided larger polymeric molecules (39). A processing method consisting of base hydrolysis followed by extraction with diethyl ether and ethyl acetate produced the highest concentration of phenolic compounds when used on apple pomace (40). Another study found that extracting pomegranate peel powder with equal volumes of ethanol and water, with a solid:solvent ratio of 1:10, for a period of 24-48 hours, was the most effective method of extracting polyphenols from pomegranates (41). The amount of polyphenolics shown in **Figure 1** (35) and their composition in the fruit varies widely even within the same species, as shown by analyzing 31 different cultivars of grapes from around the world (42).

**Table 1:** The major phenolic compounds found in cranberries, blueberries, grapes, apples, pomegranates, and oranges.

Fruit	Major phenolic compounds present
**Cranberries**	Benzoic acid, Peonidin 3-O-galactoside, Quercetin 3-O-galactoside, Peonidin 3-O-arabinoside, Cyanidin 3-O-galactoside, Quercetin 3-O-rhamnoside, Myricetin 3-O-arabinoside, Quercetin 3-O-arabinoside, Cyanidin 3-O-arabinoside, Peonidin 3-O-glucoside
**Blueberries**	5-Caffeoylquinic acid, Malvidin 3-O-glucoside, Malvidin 3-O-galactoside, Delphinidin 3-O-galactoside, Delphinidin 3-O-glucoside, Malvidin 3-O-(6”-acetyl-glucoside), Petunidin 3-O-glucoside, Petunidin 3-O-galactoside, Cyanidin 3-O-galactoside, Malvidin 3-O-arabinoside
**Grapes** **(*Vitis vinifera* L.)**	Malvidin 3-O-glucoside, Malvidin 3-O-(6”-p-courmaroyl-glucoside), Malvidin 3-O-(6”-acetyl-glucoside), Peonidin 3-O-glucoside, (+)-Catechin (-),-Epicatechin, Petunidin 3-O-glucoside, Delphinidin 3-O-glucoside, Quercetin 3-O-glucuronide, (-)-Epicatechin 3-O-gallate
**Apples** **(*Malus domestica*)**	Procyanidin dimer B2, 5-Caffeoylquinic acid, (-)-Epicatechin, Phloridzin, Phloretin 2’-O-xylosyl-glucoside, Quercetin-3-O-galactoside, 4-p-Coumaroylquinic acid, Quercetin 3-O-arabinoside, Quercetin 3-O-rhamnoside, (+)-Catechin
**Pomegranates** **(*Punica granatum* L.)**	(+)-Catechin, (+)-Gallocatechin, (-)-Epigallocatechin, Procyanidin dimer B3, Procyanidin dimer B1, (-)-Epicatechin
**Oranges** **(*Citrus sinensis* L.)**	Hesperetin, Naringenin, Lariciresinol, Pinoresinol, Secoisolaricinesinol, Syringaresinol, Kaempferol, Matairesinol, Medioresinol

The compounds presented in this list are the top ten most prevalent compounds in each type of fruit according to (35) as an average of several studies on each fruit.

### Purified Bioactives

Purified, fruit-derived polyphenols, including catechin, epicatechin, gallic acid esters, gallocatechin and epigallocatechin, have demonstrated considerable antimicrobial effects against some pathogens, including *Salmonella* which is an important foodborne pathogen associated with poultry (12, 43). The health benefits, including antioxidant activities of polyphenols in fruits and spices on humans have been reviewed in detail elsewhere (44). Positive effects on the host’s immune system have been induced using many of these compounds, both as part of a complex mixture and as purified compounds.

Gallic acid, one of the most common phenolic compounds in plants, has been studied extensively and found to have beneficial effects in treating a large range of diseases and malignancies (45). Ellagic acid, a dimerized form of gallic acid in many types of berries, has been found to inhibit aflatoxin production by fungi species (46), decrease colitis symptoms in rats (47), inhibit the growth of several bacteria species including *Helicobacter pylori* (48), *Aeromonas hydrophila* (49), *Propionibacterium acnes* and *S. aureus* (50). Tannic acid, a polymer of gallic acid, has been shown to act synergistically to decrease minimal inhibitory concentrations (MICs) of oxacillin and cefdinir against *S. aureus* to less than 0.06 mg/L, although this effect was not as pronounced on blood-agar plates (51). Additionally, another study has proposed that tannic acid decreased the integrity of the bacterial cell wall by attacking peptidoglycan and prevented biofilm formation (52). The anti-inflammatory activity of caffeic acid from cranberries has been reported in murine models of colitis (53). Extractable polyphenols, found in high concentrations in many plants, have the potential to provide a wide range of health benefits. Anti-inflammatory, immunomodulatory, and antimicrobial effects of anthocyanins have been described in human, avian, and murine infection models (54). Anthocyanins are especially prevalent in berries such as grapes and strawberries. Although pure compounds have not been studied extensively in a chicken model, many *in vitro* and *in vivo* rat or human evaluations have been conducted. In light of encouraging results, it is worth considering that these compounds could have potential beneficial effects in chickens raised without antibiotic growth promoters.

Bioactive substances in fruit may have synergistic effects when applied as a mixture, as is the case when using whole fruits or pomaces, as opposed to purified or refined extracts which may contain only a few compounds. Although pure compounds are easier to standardize and more concentrated, there may be benefits of using a less processed fruit. Containing a multitude of compounds which could work synergistically, whole fruit pomaces are less likely to induce resistance in bacteria against them. In addition, pomaces have a much lower cost of processing due to fewer purification steps. The preparation of berry pomaces and their chemical compositions have been described to reflect their potential use in poultry feed. Data from these studies reported that berry pomaces and their ethanolic extracts decreased the incidence of NE and coccidiosis when compared to bacitracin, a polypeptide antibiotic traditionally used for NE disease prevention. In addition, the ethanolic extracts induced antimicrobial activities against multiple antibiotic resistant *Salmonella* serovars isolated from broilers. In the current efforts on developing antibiotic-free and organic broiler chicken production, it seems to be difficult to access the economic benefit of using pomace as feed additive. Proper delivery methods of pomace in feed as well as its stabilization and anticaking strategies would need to be developed further. There is also a lack of knowledge of specific modes of action of fruit pomace in order to determine an effective dose in chicken.

### Modes of Action

The pleiotropic effects of fruits pomaces or extracts on immune system may be due to their multiple phenolic and non-phenolic contents. Interestingly, it has been reported that wild birds (*Sylvia atricapilla*) select their food dependent on the perceived flavonoid content, which when present in the diet were shown to increase the humoral immune response mounted by the birds (55). The presence of such a wealth of compounds, in addition to carbohydrates, proteins, lipids and minerals, make it difficult to establish exact mode of actions of fruits products on immune system (56). Various mechanisms of flavonoids on enzyme function and regulation of gene and protein expression have been reviewed (57). The possibility of flavonoids, including quercetin and pomegranate polyphenols, inducing T regulatory cells through the inhibition of mTOR (mechanistic target of rapamycin) has been explored, albeit in human cancer cells (58). If direct mechanisms can be suggested, indirect mechanisms such as those derived from metabolism of phytonutrients and shaping gut microbiota should not be ignored since gut microbiota play important role in immunity against pathogens (12, 56). Bioactive contents of fruits pomaces and extracts can be efficient free radical scavengers and iron chelators, which can in turn allow them to prevent diseases and combat pathogens (59). In humans, it has been reported that regular consumption of berries significantly reduced oxidative stress and improved the host's resistance against H_2_O_2_-induced DNA damage (60, 61). Understanding the mode of action of fruits and their extracts on poultry immunity would enhance the competitiveness and innovation within the food industry by: 1) providing comprehensive information on their use in production; 2) addressing increased public concerns about food security and safety; 3) decreasing the losses associated with poor poultry health while improving performance; and 4) finding a new application for fruit byproducts (wastes), which could be an inexpensive alternative to antibiotics.

## Fruit Byproducts in Avian Feed

Pomaces and extracts from various fruits have been investigated *in vivo* as feed additives in broiler chickens, displayed in **Tables 2** through **5**, separated by fruit. These tables do not include studies on pure extracts (for example, ellagic acid). Between the quantities of each compound present, and possible synergistic effects of multiple chemicals, the administration of a purified compound versus raw extracts or whole fruit is different enough to make a direct comparison possibly misleading. Not every study listed in these Tables presented data on both the immune system and gut microbiota, therefore some metrics may be absent for certain studies. Studies directly measuring immune parameters would make comparison easier and should be undertaken. For the sake of brevity, only statistically significant results are mentioned within the Tables. Comparisons were made with control birds in each study unless otherwise noted. There are more studies available in the literature on carcass quality, meat preservation, and growth performance using a variety of fruits such as strawberry (78–80), however these were not included as meat quality and economic performance are not the focus of this paper.

**Table 2:** A comparison of *in vivo* feeding studies of blueberry byproducts and extracts in poultry, highlighting significant effects on the immune system and gut microbiota in live chickens.

Type of plant extract	Dose & method of administration	Type of poultry (sample size)	Effects on immunity	Effects on gut microbiota	Reference
**Blueberry (lowbush) pomace**	1% and 2%, in feed, applied between 7 and 21 days of age	Cobb 308 broilers (500)	ND	Increased aerobic Gram-positive species (day 29 and 42). Decreased aerobic Gram-positive species (day 64), *Lactobacillus* (day 64), *Enterococcus* (day 21 and 64)	(62)
**Blueberry pomace**	1% and 2% in feed, applied between 0 and 30 days of age	Cobb 500 male broilers (2,800)	Lower frequency of intestinal lesions compared to bacitracin-treated birds	Higher *C. perfringens*, *E. coli*, and *Lactobacillus* counts in ceca.	(63)
**Blueberry pomace, 80% ethanol extract**	150 ppm and 300 ppm in feed, applied between 0 and 30 days of age	Cobb 500 male broilers (2,800)	Similar rates of necrotic enteritis lesions to control birds.	Higher *C. perfringens* and *Lactobacillus* counts in ceca. 300 ppm treatment increased amounts of *Acidobacteria*.	(63)

ND, Not determined.

**Table 3:** A comparison of *in vivo* feeding studies of cranberry byproducts and extracts in poultry, highlighting significant effects on the immune system and gut microbiota in live chickens.

Type of plant extract	Dose & method of administration	Type of poultry (sample size)	Effects on immunity	Effects on gut microbiota	Reference
**Cranberry pomace**	1% and 2% in feed, applied between 0 and 30 days of age	Cobb 500 male broilers (2,800)	Lower frequency of intestinal lesions compared to bacitracin-treated birds. Significantly upregulated CCR6, CD14, CSF2, JAK2, TLR15, HMBS, IL-4 and IL-5 in spleen	Lowered *C. perfringens* and *E. coli* counts in ceca, higher *Lactobacillus* abundance.	(63)
**Cranberry pomace, 80% ethanol extract**	150 ppm and 300 ppm in feed, applied between 0 and 30 days of age	Cobb 500 male broilers (2,800)	Significantly upregulated CSF2, IL-4, IL-5, TLR15 in spleen.	Lowered *C. perfringens* and *E. coli* counts in ceca, higher *Lactobacillus* abundance for the 150 ppm treatment. Lower *E. coli* and *Lactobacillus* while higher *C. perfringens* for the 300 ppm treatment.	(63)
**Cranberry, Non-dialyzable materials**	Orally administered liquid, 2, 4, or 8 mg/mL, applied between 7 and 11 days of age	Ross 308 male broilers (1,200)	*In vitro:* chicken heterophil phagocytosis increased. *In vivo*: IgM levels increased on day 35	Did not kill *S. aureus in vitro*, however the bacteria was more susceptible to phagocytosis by chicken heterophils.	(64)
**Cranberry pomace, EtOH fractions**	1% and 2% in feed, applied between 8 and 36 days of age	Cobb 500 broilers (600)	ND	Slow increase in *Euryarchaeota* populations over time in treated birds vs control. Lower levels of *Fusobacteria* and higher levels of *Bifidobacterium, Ruminococcus*, & *Oscillospira* associated with pomace.	(65)
**Cranberry fruit extract**	40, 80, 160 mg/kg of feed, applied between 0 and 35 days of age	Ross 308 broilers (1,200)	No significant effects on intestinal or general health were noted.	*Enterococcus* abundance was lower in birds receiving 80 mg/kg of feed cranberry extract. No difference in *Lactobacillus* between treatments.	(66)

ND, Not determined.

**Table 4:** A comparison of *in vivo* feeding studies of grape byproducts and extracts in poultry, highlighting significant effects on the immune system and gut microbiota in live chickens.

Type of plant extract	Dose & method of administration	Type of poultry (sample size)	Effects on immunity	Effects on gut microbiota	Reference
**Grape seeds**	10, 20, 40 g/kg of feed, applied between 1 and 42 days of age	Cobb 500 broilers (300)	ND	Higher *Lactobacillus*, lower *E. coli* and *Streptococcus* counts in every grape seed treatment.	(67)
**Red grape pomace concentrate**	15, 30, and 60 g/kg of feed, applied between 21 and 42 days of age	Cobb male broilers (180)	Antioxidant activities in ileal content and excreta was significantly higher than control.	ND	(68)
**Red grape pomace**	5% and 10%, additional groups with these concentrations supplemented with enzymes	Cobb male broilers (300)	Plasma antioxidant activity was much higher in treated birds.	5% pomace reduced *C. perfringens* counts. *Lactobacillus, E. coli* and *Enterobacteriaceae* were not affected.	(69)
**Grape extract**	2.5 g/kg and 5 g/kg of feed, applied between 1 and 21 days of age	Cobb male broilers (105)	Intestinal goblet cells and mucin production (intestinal barrier) were not affected. Sialic acid decreased.	Lactic acid bacteria and *E. coli* populations were decreased by the extract. *C. perfringens* was not affected. *Enterobacteriaceae* decreased slightly.	(70)
**Grape seed extract**	125, 250, 500, 1000, and 2000 ppm in feed from 0 to 42 days of age	Ross 308 broilers (245)	Increase of NDV-specific antibodies by up to 57% at day 28 and 76% at day 35. Significantly increased antioxidant status of the liver tissue	ND	(13)
**Grape seed extract**	5, 10, 20, 40, and 80 mg/kg in feed applied between days 1 and 15	Shiqizha broilers (216)	After *E. tenella* infection, mortality was 46% in the control group and 6.7% in the 10 mg/kg GSE group. Lesion scores in all GSE groups were significantly better than the control.	ND	(71)
**Fermented red grape skin**	30 and 60 g/kg in feed applied between days 1 and 21	Cobb male broilers (150)	ND	No effect on *Clostridium* spp. or lactic acid bacteria.	(72)
**Unfermented red grape skin**	30 and 60 g/kg in feed applied between days 1 and 21	Cobb male broilers (150)	ND	No effect on *Clostridium* spp. or lactic acid bacteria. Lower numbers of *E. coli* compared to fermented extract.	(72)
**Grape pomace concentrate**	60 g/kg of feed, applied from days 1-21	Cobb male broilers (100)	ND	In ileum: *Enterococcus* spp. increased, *Clostridium* spp. decreased, no difference in *E. coli* counts compared to control. *Lactobacillus* decreased. In ceca: *Enterococcus, E. coli, Lactobacillus, Clostridium* significantly higher. Higher biodiversity in ceca.	(73)
**Grape seed extract**	7.2 g/kg of feed, applied from days 1-21	Cobb male broilers (100)	ND	In ileum: *Enterococcus* spp. increased, *Clostridium* spp. decreased, no difference in *E. coli* counts compared to control. In ceca: *Enterococcus, E. coli, Lactobacillus, Clostridium* significantly higher. *E. coli* and *Enterococcus* were higher than the grape pomace concentrate treatment. Higher biodiversity in ceca.	(73)
**Muscadine grape pomace**	0.5%, 2% and 5% feed weight, applied from day 12 - 42	Broiler chicks (960)	Unvaccinated birds dosed with 2% and 5% pomace were significantly more resistant to the development of necrotic enteritis lesions after dosing with *Eimeria* spp. and *Clostridium perfringens.* Vaccinated and challenged birds were also more resistant to infection after 0.5% and 2% pomace treatments.	ND	(74)

NDV, Newcastle Disease Virus; ND, Not determined.

**Table 5:** A comparison of *in vivo* feeding studies of various other fruit byproducts and extracts in poultry, highlighting significant effects on the immune system and gut microbiota in live chickens.

Type of plant extract	Dose & method of administration	Type of poultry (sample size)	Effects on immunity	Effects on gut microbiota	Reference
**Green tea and pomegranate extract**	2 mL/L in water, applied on days 0-4, 10,11,20, and 21	Ross 308 male broilers (480)	ND	Bacilli, Lactobacillales, Lactobacillaceae, Peptococcaceae, *Roseburia* significantly higher in treatment group. *Shuttleworthia* was lower in treatment group.	(75)
**Orange peel extract**	1000 and 1250 ppm in water, applied from day 1 - 42	Ross-308 male broilers (300)	Serum IgG and IgM titers were elevated, antibody titers against NDV and AI were increased, and percentage of heterophils was lower in treatment groups.	ND	(76)
**Dried apple pomace**	4, 8, 12, 16, and 20% in feed applied from day 1 - 42	Ross-308 broiler chicks (480)	Higher antibody titers against NDV and AI in some treatment groups, but not all	ND	(77)

NDV, Newcastle Disease Virus; AI, Avian Influenza; ND, Not determined.

### Berries

A summary of studies performed on blueberry products can be found in **Table 2**, and cranberry products in **Table 3**. Although the immune-modifying compounds of lowbush blueberry pomace were not investigated, the pomace was found to significantly decrease concentrations of blood metabolites such as serum glucose and triglycerides in 21-day old slow growth broilers raised on pasture (62). It is worth noting that this study applied the blueberry pomace treatment for two weeks in 7-day old chicks, before transferring them to a pasture-style environment at 21 days of age. A study with non-dialyzable materials containing high-molecular weight compounds of cranberry juice enhanced phagocytic functions of chicken heterophils against *S. aureus*, as well as some humoral response in broilers (64). A subsequent study by the same group on cranberry pomace feeding during a longer administration period found similar effects on lowering levels of serum glucose and triglycerides while increasing blood serum iron level (65). A recent study evaluated different types of cranberry and blueberry products, including pomaces and their ethanolic extracts, and found promising results with regards to the intestinal health of the birds as stated above (63). The blood metabolites in this study once again agreed with previous studies, namely that triglycerides were significantly lower in fruit-treated birds, although iron was not significantly affected. They also found significant differences in cytokine gene expression in chickens fed cranberry products. Notably, the modulated cytokines included mostly Th2 cytokines, which help to regulate antibody-mediated immune responses *via* Th2 cells. Analysis of immunoglobulin levels as well as liver and bursal gene expression, revealed that dietary cranberry products increased the serum IgY level, modulated the innate immune and suppressed proinflammatory cytokines in 21-day old broilers (81).

### Grapes

A summary of studies performed on grape products can be found in **Table 4**. When grape pomace concentrate was compared to grape seed extract (73), the levels of extractable polyphenols were standardized between the two different products, however the results between the two still differed, suggesting that there are other factors influencing microbiota besides exclusively polyphenols. Studies that measured the level of polyphenolic compounds in the blood plasma or ileal digesta consistently found that the concentration of polyphenols was higher in the tissues of treated birds vs untreated birds. This could contribute to a health benefit for consumers who eat these meats, as these compounds are regarded to have health benefits for humans in small quantities (82). Higher levels of polyphenols generally lead to higher levels of antioxidants, and therefore a greater resistance to oxidative stresses. Although all of the studies agreed on this, they did not agree on many other parameters: whether certain species of bacteria in the ileum and ceca of the birds were increased or decreased, and whether antibody or resistance levels were increased or not significantly affected. With the large variety of grapes available, all containing different levels of polyphenolics and other compounds, it can be difficult to compare two different studies that used different fruits – especially since many of the studies did not name the cultivar of grape used, the locality it was harvested in, or even whether the grape was white or red. The disparity between different cultivars of grapes, especially when grouped by geographical origin, is significant (42).

### Apples, Pomegranates, and Other Fruits

**Table 5** contains a summary of studies performed on other fruits, including apples, pomegranates, and oranges. Apple is an important commercial crop for many countries. In poultry it has been suggested that apple byproducts could be included up to 5% in diets for broilers and up to 10% for laying hens diet to reduce oxidative stress (83). It has been reported that inclusion of up to 12% dry apple pomace in diet increased antibody levels against some pathogenic virus in poultry (77). However, this group also found that inclusion levels of 12 – 20% lowered the feed intake of birds, which lead them to suggest this may have been caused by the high fiber content of this food source.

Pomegranates have also been used in feed and drinking water. The addition of pomegranate rind extract and green tea to broilers’ water during critical periods in development showed a significant increase in the population of bacilli, specifically Lactobacilli, in the caeca (75). Another study compared pomegranate peel, pomegranate peel extract, and purified α-tocopheryl acetate, and found a significantly higher antioxidant activity in most of the treated groups, with the exception of the birds treated with the lowest concentration of pomegranate peel (84). These authors also reported that the pomegranate peel extract and the α-tocopheryl increased the amount of polyunsaturated fatty acids in the breast meat. Supplementation with pomegranate peel extract or α-tocopheryl did not impact the performance of the birds, however the pomegranate peel decreased the body weight gain, feed intake, and feed efficiency of the birds, overall making it the least desirable treatment (85).

A study on orange peel extracts added to water found that broilers vaccinated against various diseases had elevated antibody titers against these diseases in a dose-dependent manner (76). Although this cannot be directly compared to studies on other fruits, due to the dissimilarity in both the species of fruit and the method of administration, it is worth noting this positive result.

### Potential Side Effects

Although there are many positive effects of plant-derived phenolic materials, some of them demonstrated negative effects as well. For example, the sensory profile of meat from chickens fed 5% grape seed meal was found to have a less sweet and more metallic flavor, while being more stringy, but overall the diet did not significantly impact the meat acceptability (86). A study on various pure extracts in poultry feed determined that anthocyanins from cherries added to poultry feed significantly increased the daily feed intake, but resulted in a slightly lower average weight, which translates to a lower feed efficiency (87). The addition of pomegranate peel into the feed has been shown to impair body weight gain, feed intake, and feed efficiency (85). Adding more than 5% dried apple pomace to feed caused birds to lose weight (83). It is therefore not advisable to feed these additives in high concentrations without fully discerning the effects on both the living chicken and the carcass or meat quality.

## Conclusion

Immune stimulation and modulation are becoming increasingly necessary in the fight against pathogens. Several options for immunomodulation have been explored, and each has its own advantages and disadvantages. While there are some substantial limitations of studying and implementing fruit by-products as feed additives, there are also many potential benefits for the immune system, thereby increasing resistance to disease. This makes fruit wastes not only inexpensive alternatives to traditional antibiotics, but value-added and environmentally friendly products that promote the circular economy. Many types of fruit contain high levels of desirable polyphenolic and antioxidant compounds. However, a balance must be struck between minimal processing (inexpensive, large volumes produced, low levels of many compounds) and high levels of processing (expensive, small volumes produced, high levels of a few targeted compounds). More studies are needed on the effects of these byproducts and their associated extracts. Additionally, the type of chicken, farm setup, feed composition, vaccination schedule, treatment schedule and dosage, as well as the parameters being evaluated need to be standardized in order for studies to be comparable. There has not yet been a study that evaluates every parameter of a certain product (immune robustness, growth performance, feed conversion ratio, resistance to disease, gut microflora, meat nutrition, sensory profile, and shelf-life). Once more is known about one type of fruit waste, comparisons to other types may be made and the most effective can be determined. This review indicates the potential of fruit products as feed supplements to enhance the immunity in birds, providing information for future investigations to develop them in poultry feeding strategies.

## Author Contributions

MSD conceived the works and provided resources. TH found available literatures and wrote the paper. MSD, PB and SS provided guidelines, reviewed and edited the manuscript. All authors contributed to the article and approved the submitted version.

## Funding

The research has been funded through the A-base program by Agriculture and Agri-Food Canada (PSS #2574, J-001788).

## Conflict of Interest

The authors declare that the research was conducted in the absence of any commercial or financial relationships that could be construed as a potential conflict of interest.
